# Supplementary material for: A terminal selector prevents a Hox transcriptional switch to safeguard motor neuron identity throughout life
Source: eLife. 2020 Jan 3;9:e50065. doi: 10.7554/eLife.50065 (PMC6944445; doi:10.7554/eLife.50065)
Supplement: Supplementary file 1. [file elife-50065-supp1.docx]

**Supplementary File 1:**

**UNC-3 binding sites (COE motifs) are not found in the *cis*-regulatory region of VD- and VC-expressed terminal identity genes.**

| **Terminal identity gene** | **Expression** | **Effect in *unc-3 (-)*** | **COE motif** |
| --- | --- | --- | --- |
| *unc-17* | Cholinergic MNs | Loss of expression in MNs | Yes |
| *cho-1* | Cholinergic MNs |  | Yes |
| *acr-2* | Cholinergic MNs |  | Yes |
| *del-1* | Cholinergic MNs |  | Yes |
| *unc-129* | Cholinergic MNs |  | Yes |
| *nca-1* | Cholinergic MNs |  | Yes |
| *slo-2* | Cholinergic MNs |  | Yes |
|  |  |  |  |
| *ser-2* | VD | Ectopic expression in MNs | No |
| *oig-1* | VD |  | No |
| *flp-11* | DD/VD/VC |  | No |
| *twk-46* | DD/VD/VC |  | No |
| *ilys-4* | DD/VD/VC |  | No |
| *glr-5* | VC |  | No |
| *ida-1* | VC |  | No |
| *srb-16* | VC |  | No |
