# Supplementary material for: A terminal selector prevents a Hox transcriptional switch to safeguard motor neuron identity throughout life
Source: eLife. 2020 Jan 3;9:e50065. doi: 10.7554/eLife.50065 (PMC6944445; doi:10.7554/eLife.50065)
Supplement: Supplementary file 2. — Asterisk (*) highlights novel LIN-39 targets; N. D: Not Determined. The selected cis-regulatory regions are LIN-39 ChIP-seq peaks that fall within the DNA sequence used for our reporter gene constructs (except for del-1). The UNC-3 binding sites (COE motifs 23 bp) have been previously described in Kratsios et al. (2012). The LIN-39 binding sites were predicted by a FIMO search (p<0.005). The UNC-30 binding site on ser-2 locus was predicted by a FIMO search. The UNC-30 site on oig-1 was experimentally validated in Howell et al. (2015). [file elife-50065-supp2.docx]

| **LIN-39 targets in cholinergic MNs** | | | |  | **LIN-39 targets in GABAergic VD MNs** | | | | |
| --- | --- | --- | --- | --- | --- | --- | --- | --- | --- |
|  | **COE** motif (relative to ATG) | **LIN-39** sites  (relative to ATG) | **P value** |  |  | | **UNC-30** sites  (relative to ATG) | **LIN-39** sites  (relative to ATG) | **P value** |
| *unc-129* | *COE1 (-262-240)*  *COE2 (-346-324)*  *COE3 (-458-436)* | *LIN-39 sites:*  *#1 (-444-437)*  *#2 (-421-414)*  *#3 (-376-369)* | *#1 p=0.000119*  *#2 p=0.00474*  *#3 p=0.0014* |  | *oig-1** | *Peak#4* | *N. D.* | *LIN-39 sites:*  *#1 (-1957-1950)*  *#2 (-1868-1861)*  *#3 (-1804-1797)* | *#1 p=0.00162*  *#2 p=0.00144*  *#3 p=0.000645* |
|  |  |  |  |  |  | *Peak#3* | *UNC-30 site:*  *(-955-951)* | *#4 (-974-967)*  *#5 (-843-836)*  *#6 (-801-794)* | *#4 p=0.00377*  *#5 p=0.00474*  *#6 p=0.00441* |
| *del-1* | *COE1 (-194-172)*  *COE2 (-2374-2352)* | *LIN-39 sites:*  *#1 (-276-269)*  *#2 (-323-316)*  *#3 (-389-382)*  *#4 (-487-480)*  *#5 (-2445-2438)*  *#6 (-2609-2602)* | *#1 p=0.00259*  *#2 p=0.00386*  *#3 p=0.00187*  *#4 p=0.00402*  *#5 p=0.0000396*  *#6 p=0.00483* |  | *ser-2** | | *UNC-30 site:*  *#1 (-253-246)* | *LIN-39 site:*  *#1 (-311-304)*  *#2 (-254-247)*  *#3 (-198-191)*  *#4 (-183-176)*  *#5 (-139-132)*  *#6 (+121-128)*  *#7 (+141-148)* | *#1 p=0.00368*  *#2 p=0.00274*  *#3 p=0.00472*  *#4 p=0.00181*  *#5 p=0.00144*  *#6 p=0.00461*  *#7 p=0.000491* |
| *acr-2** | *COE2 (-483-461)*  *COE1 (-147-126)* | *LIN-39 sites:*  *#1 (-791-784)*  *#2 (-759-752)*  *#3 (-646-639)*  *#4 (-580-564)*  *#5 (-486-479)*  *#6 (-482-475)*  *#7 (-468-461)*  *#8 (-464-457)* | *#1 p=0.000864*  *#2 p=0.000574*  *#3 p=0.0016*  *#4 p=0.0047*  *#5 p=0.00205*  *#6 p=0.00135*  *#7 p=0.0045*  *#8 p=0.00325* |  |  | |  |  |  |
| *dbl-1** | *COE1 (-1433-1411)*  *COE2 (-1231-1210)* | *LIN-39 sites:*  *#1 (-1475-1468)*  *#2 (-1460-1453)*  *#3 (-1217-1210)*  *#4 (-1209-1202)*  *#5 (-1087-1080)*  *#6 (-1078-1071)*  *#7 (-1035-1-28)* | *#1 p=0.00474*  *#2 p=0.00441*  *#3 p=0.000947*  *#4 p=0.00199*  *#5 p=0.000513*  *#6 p=0.0027*  *#7 p=0.000574* |  |  | |  |  |  |

**Supplementary File 2:**

**LIN-39/Hox targets in cholinergic and GABAergic (VD) motor neurons.**
